# Megaconial congenital muscular dystrophy due to novel CHKB variants: a case report and literature review

**DOI:** 10.1186/s13395-022-00306-8

**Published:** 2022-09-29

**Authors:** Francesca Magri, Sara Antognozzi, Michela Ripolone, Simona Zanotti, Laura Napoli, Patrizia Ciscato, Daniele Velardo, Giulietta Scuvera, Valeria Nicotra, Antonella Giacobbe, Donatella Milani, Francesco Fortunato, Manuela Garbellini, Monica Sciacco, Stefania Corti, Giacomo Pietro Comi, Dario Ronchi

**Affiliations:** 1 IRCCS Fondazione Ca’ Granda Ospedale Maggiore Policlinico, Neurology Unit, Milan, Italy; 2 Dino Ferrari Center, Department of Pathophysiology and Transplantation, University of Milan, Milan, Italy; 3 IRCCS Fondazione Ca’ Granda Ospedale Maggiore Policlinico, Neuromuscular and Rare Disease Unit, Milan, Italy; 4 IRCCS Fondazione Ca’ Granda Ospedale Maggiore Policlinico, Medical Genetics Unit, Woman-Child-Newborn Department, Milan, Italy; 5 IRCCS Fondazione Ca’ Granda Ospedale Maggiore Policlinico, Neonatal Intensive Care Unit, Milan, Italy

**Keywords:** Choline kinase beta (CHKB), Megaconial congenital muscular dystrophy, Enlarged mitochondria, Mitochondrial dynamics

## Abstract

**Background:**

Choline kinase beta (CHKB) catalyzes the first step in the de novo biosynthesis of phosphatidyl choline and phosphatidylethanolamine via the Kennedy pathway. Derangement of this pathway might also influence the homeostasis of mitochondrial membranes.

Autosomal recessive *CHKB* mutations cause a rare form of congenital muscular dystrophy known as megaconial congenital muscular dystrophy (MCMD).

**Case presentation:**

We describe a novel proband presenting MCMD due to unpublished *CHKB* mutations. The patient is a 6-year-old boy who came to our attention for cognitive impairment and slowly progressive muscular weakness. He was the first son of non-consanguineous healthy parents from Sri Lanka. Neurological examination showed proximal weakness at four limbs, weak osteotendinous reflexes, Gowers’ maneuver, and waddling gate. Creatine kinase levels were mildly increased. EMG and brain MRI were normal. Left quadriceps skeletal muscle biopsy showed a myopathic pattern with nuclear centralizations and connective tissue increase. Histological and histochemical staining suggested subsarcolemmal localization and dimensional increase of mitochondria. Ultrastructural analysis confirmed the presence of enlarged (“megaconial”) mitochondria. Direct sequencing of *CHKB* identified two novel defects: the c.1060G > C (p.Gly354Arg) substitution and the c.448-56_29del intronic deletion, segregating from father and mother, respectively. Subcloning of RT-PCR amplicons from patient’s muscle RNA showed that c.448-56_29del results in the partial retention (14 nucleotides) of intron 3, altering physiological splicing and transcript stability. Biochemical studies showed reduced levels of the mitochondrial fission factor DRP1 and the severe impairment of mitochondrial respiratory chain activity in patient’s muscle compared to controls.

**Conclusions:**

This report expands the molecular findings associated with MCMD and confirms the importance of considering *CHKB* variants in the differential diagnosis of patients presenting with muscular dystrophy and mental retardation. The clinical outcome of MCMD patients seems to be influenced by *CHKB* molecular defects. Histological and ultrastructural examination of muscle biopsy directed molecular studies and allowed the identification and characterization of an intronic mutation, usually escaping standard molecular testing.

**Supplementary Information:**

The online version contains supplementary material available at 10.1186/s13395-022-00306-8.

## Background

Megaconial congenital muscular dystrophy (MCMD) is a rare neuromuscular disorder [1] due to recessive mutations in *CHKB* [2], encoding choline kinase beta. This enzyme takes part in the de novo biosynthesis of phosphatidyl choline and phosphatidylethanolamine (Kennedy pathway) which are important lipid components of the cellular membranes, including those found in mitochondria [3].

Main clinical features of MCMD include neonatal hypotonia and developmental delay without brain malformation and neuromuscular involvement characterized by slowly progressive proximal weakness. The cognitive involvement can resemble Rett syndrome [4] and early mortality can be the consequence of cardiomyopathic involvement.

Although muscle biopsies often reveal the peculiar absence of mitochondria in the centre of fibers and enlarged (“megaconial”) mitochondria in the peripheries, signs of mitochondrial dysfunction have been sporadically investigated in patients [5, 6].

We describe a new case of MCMD in a young boy presenting with intellectual and motor delay. Muscle biopsy prompted the analysis of *CHKB* and was fundamental for the validation of the identified variants. Protein and biochemical studies linked altered mitochondrial dynamics to mitochondrial dysfunction.

## Case presentation

### Clinical and pathological features

An 18-month-old boy came to our attention presenting intellectual and motor delay. He was the first son of healthy non-consanguineous parents coming from Sri-Lanka and had unremarkable antenatal and perinatal history.

At 18 months of age spontaneous speech was absent and the highest motor milestone achieved was the sitting position. ENT evaluation was normal, excluding deafness as a potential cause of speech delay. At clinical evaluation, café au lait spots were noticed, but ophthalmology and dermatological evaluations were normal.

The motor function slightly improved in the following years: the patient started to walk independently at 2 years of age and at 4 years of age neurological examination showed mild proximal weakness at four limbs, with Gowers’ maneuver and weak osteotendinous reflexes. Cranial nerves were normal, as well as coordination and sensibility. The cognitive delay was still severe since the patient was not able to speak, even if he presented partial comprehension and good nonverbal communication.

Electromyography showed a normal pattern. Creatine kinase (CK) levels ranged from normal to mildly increased (560 U/L, reference range = 38–174 U/L). Brain magnetic resonance, performed at 6 years of age, showed focal thinning of corpus callosum isthmus and mild asymmetry of temporal horn of lateral ventricles.

Cardiac evaluation (electrocardiogram and echocardiogram) and respiratory assessment were normal. Mild dysmorphic features such as slight frontal drafts and wide nasal bridge were evident and CGH array was performed showing a 2.5-Mb deletion at chromosome Yp11.2. This deletion was present also in the healthy proband’s father excluding the pathogenic role of this finding.

At 6 years of age cognitive involvement was unchanged, with absent verbal expression, social interaction and communication difficulties, aggressive behavior, repetitive habits, restricted interests, and lack of reciprocity. Neuromuscular evaluation showed a mild worsening of muscular weakness predominantly at lower limbs, with waddling gait and difficulties in standing from a chair.

**Fig. 1:**
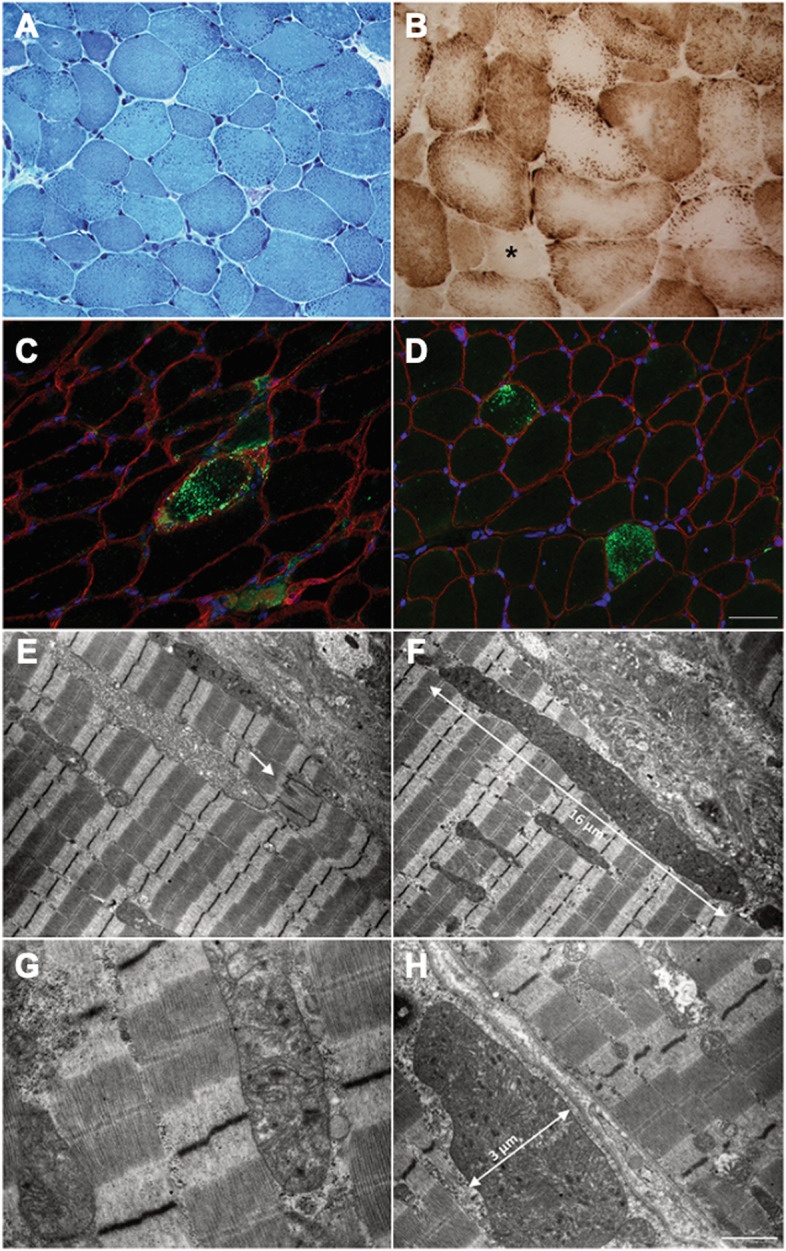
Histological, immunohistochemical, and ultrastructural studies in patient’s muscle.** A** Light microscopy observations of MTG showed moderately increased variation in fiber size with a consistent number of hypotrophic fibers and the presence of abnormal subsarcolemmal granules in several fibers. **B** Staining for COX activity shows the presence of large mitochondria at the periphery of fibers and central areas lacking any activity (* indicates lack of COX activity due to mitochondrial depletion in those areas/fibers). Immunofluorescence staining for p62 (**C**) and LC3A (**D**) was positive in some fibers. Caveolin-3 was used for membrane staining and nuclei were counterstained with DAPI. **E–H** Representative images of the main alterations in skeletal muscle and in mitochondria morphology. **E** Minor abnormalities of the of Z-line (indicated by an arrow), mitochondrial alterations including giant mitochondria (**F**), mitochondria with globular inclusions (**G**), and swollen mitochondria (**H**). Scale bars: A, 400 µm; B**–**D, 250 µm, E**–**F 2.27 µm; G, 0.83 µm; H, 1.43 µm

The patient underwent muscular biopsy on left quadriceps muscle. Microscopic examination revealed moderate fiber size variability with a consistent number of hypotrophic fibers (Fig. 1A). The outstanding feature of muscle biopsy, however, was the presence of abnormal subsarcolemmal mitochondria in several fibers, a feature confirmed by histochemical analysis of COX activity which showed large mitochondria mainly located at the periphery of fibers and central areas without any activity. Rare fibers completely lacked COX activity due to mitochondrial depletion (Fig. 1B).

Immunofluorescent evaluation of autophagy markers showed the presence of some fibers with intense positivity for p62 (Fig. 1C) and LC3 (Fig. 1D), suggesting activation of autophagy.

At ultrastructural examination, abnormalities of the myofibrillar architecture with Z line streaming were noted (Fig. 1E). In addition, the presence of giant mitochondria was confirmed. Some of them were enlarged in both length and diameter (Fig. 1H), also, we observed longitudinally cut mitochondria spanning the length of 5–6 sarcomeres (Fig. 1F). Mitochondria often contained electrodense globular inclusions (Fig. 1G). Smaller mitochondria with a swollen aspect displaying disorganised or broken cristae were also evident (Fig. 1H).

### Molecular and biochemical studies

**Fig. 2:**
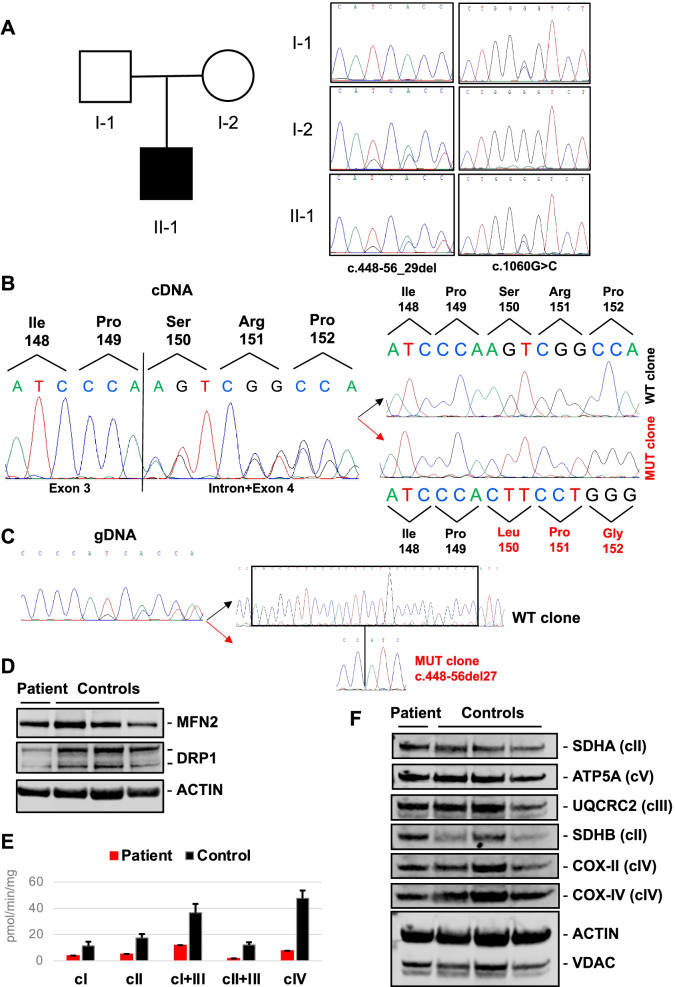
Genetic and biochemical findings. **A** Pedigree of the family investigated. Electropherogram showing the *CHKB* nucleotide substitutions detected in our patient and segregation analysis in his unaffected parents. **B** Transcript analysis of *CHKB* in patient’s muscle. Electropherograms show the sequences of RT-PCR amplicons obtained before (left) and after (right) subcloning. Subcloning experiment confirmed the partial intronic retention in CHKB ORF in patient’s muscle. **C** Sequence electropherograms of the intronic region between CHKB exons 3 and 4 and subcloning experiments confirming the the presence of a small deletion. **D** Western blot analysis of Mitofusin 2 (MFN2) and dynamin-related protein 1 (DRP1) protein levels in muscle samples from patient and controls. Actin levels were used as reference. **E** Graph showing respiratory chain enzymatic activities in Patient’s muscle compared to control levels assessed through spectrophotometric analysis (experiment in duplicate). Values are normalized to citrate synthetase (CS) activity and expressed as pmol/min/mg protein. **F** Western blot analysis of representative subunits of mitochondrial respiratory chain. No difference was observed between patients’ and control muscle samples. Actina and porin (VDAC) levels were used as reference

Direct sequencing of *CHKB* exonic regions in the patient revealed the heterozygous single nucleotide variation c.1060G > C likely resulting in the p.Gly354Arg, which was confirmed in his father (Fig. 2A). The c.1060G > C substitution has been reported in a single South Asian female subject in the gnomAD database (allele frequency 0.000004036) and it has never been related to disease. The variant was predicted as pathogenic by multiple tools including SIFT, Polyphen, Mutation Taster, Mutation Assessor, and CADD (score = 28) and was classified as a class 3 variant according ACMG guidelines.

Looking for a molecular defect on the second allele, we extracted and retrotranscribed RNA from patient’s muscle biopsy. By sequencing RT-PCR amplicons we detected a rearrangement at the junction between exons 3 and 4 which was compatible with the partial retention of a small intronic region in *CHKB* transcript. Subcloning experiments confirmed a 14-nt insertion resulting in the frameshift variant p.Ser150Leufs*8 (Fig. 2B). Therefore, we sequenced the intron comprised between exon 3 and 4 in patient’s genomic DNA and we disclosed a heterozygous deletion whose boundaries were confirmed after cloning of the genomic fragment (Fig. 2C). The identified c.448-56del27 microdeletion is predicted to alter the canonical exon 4 acceptor splice site, favoring an alternative splicing with the intronic retention of 14 nucleotides. This variant was also detected in the patient’s mother (Fig. 2A). RT-PCR analysis on blood-extracted RNA from patient’s mother (not shown) revealed the same effect detected in patient’s muscle, supporting the pathogenic role of this novel allele.

The levels of Mitofusin 2, an outer mitochondrial membrane protein known to modulate mitochondrial fusion, were unchanged comparing muscle biopsies from the patient and control subjects. Conversely, we observed a marked loss of the mitochondrial fission factor DRP1 in patient’s muscle (Fig. 2D). Spectrophotometric analysis of respiratory chain complex activities disclosed a severe multi-complex defect in the patient’s, but not in controls’, muscle samples (Fig. 2E). No difference in the steady-state protein levels of representative subunits of mitochondrial respiratory chain complexes was observed between patient’s and controls’ muscle samples (Fig. 2F).

## Discussion

**Fig. 3:**
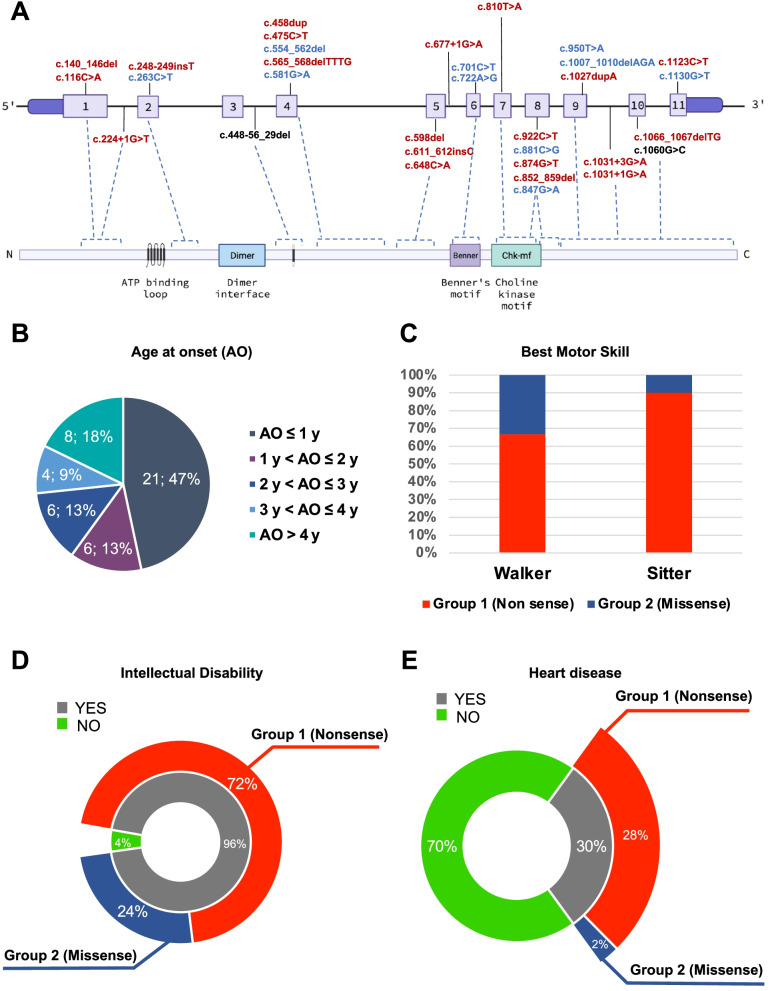
Genotype–phenotype correlation in *CHKB-*mutated patients.** A** Scheme of *CHKB* gene (above) and the encoded choline kinase beta (bottom). Mutations identified in MCMD patients are indicated in red (group 1: non-sense/frameshift mutations), blue (group 2: missense/in-frame mutations) or black (mutations disclosed in our proband). **B** Distribution of age at onset (in years) in MCMD patients. **C** Relative comparison of best motor achievements in MCMD patients stratified according to genotype. **D** Prevalence of intellectual disability in MCMD patients stratified according to genotype. **E** Prevalence of heart disease (mainly dilated cardiomyopathy) in MCMD patients stratified according to genotype

This study describes a new case of megaconial congenital muscular dystrophy (MCMD) due to two novel *CHKB* variants (Fig. 3A).

MCMD has been described for the first time as a distinct muscle pathology in 1998 by Nishino and colleagues [1]. The genetic defect underlying MCMD was disclosed in 2011 with the identification of biallelic *CHKB* mutations in 15 MCMD patients displaying early-onset muscle weakness, mental retardation, and giant mitochondria at muscle biopsy analysis [2]. Phenotypic and histological overlap was observed with the spontaneous mutant rmd (“rostrocaudal muscular dystrophy”) mouse, which exhibited congenital muscular dystrophy and identical muscle fiber pathology [7, 8].

Although MCMD might display heterogenous multisystemic involvement, hypotonia and developmental delay without brain malformations associated to neuromuscular involvement characterized by slowly progressive proximal muscle weakness is the predominantly observed phenotype. Onset of symptoms is usually within the first 4 years of age (> 80% of cases described, Fig. 3B), but MCMD can also become manifest during adolescence. The cognitive involvement can resemble Rett syndrome [4] and early mortality is usually related to heart failure consequent to dilated cardiomyopathy. Ichthyotic skin changes, and seizures have been also frequently reported (30% of subjects). In few cases, worsening after stress/illness was reported [9–11].

So far, 45 patients from 40 independent families with documented *CHKB* mutations have been described (Supplementary Table 1) [3]. More than 60% of patients described are from Asia and 16 independent cases are of Turkish origin. At least 31 different mutations in *CHKB* were reported (Supplementary Table 1 and Fig. 3A). Most of them are nonsense mutations (8 stop codon and 8 frameshift variants), followed by missense (*n* = 10) and splice-site variants (*n* = 5). Thirty-five of 40 independent probands displayed homozygous defects supporting consanguinity in their unaffected parents.

We reviewed the clinical findings in patients reported so far (*n* = 45, Supplementary Table 1). Patients were divided into two groups based on their genotype. Group 1 included 33 patients whose pathogenic variants are expected to result in the formation of only truncated messenger RNA (mRNA), which is likely to be subject to nonsense-mediated decay (premature terminations codons, splice-site variants, or a combination of both). Group 2 included the remaining 12 patients whose variants are not expected to result in the formation of truncated mRNA (missense mutations or in-frame deletions).

There was a statistically significant difference (Mann–Whitney *U* test, *p* = 0.03662) in the mean age at onset between the two groups (group 1 2.00 ± 2.03 years, range from birth to 8 years of age, median age 12 months; group 2 6.80 ± 6.95 years, range from 1 month to 17 years of age, median age 3.5 years).

Mean age at walking was 2.45 ± 1.09 years for group 1 compared with 1.66 ± 0.70 years for group 2 (Mann–Whitney *U* test, *p* = 0.02574). Eight patients from group 1 (patients 6, 9, 11, 14, 17, 20, 29, and 30) never acquired independent ambulation [2, 4, 8, 12–15]. In group 2 unassisted ambulation was achieved in all patients with the exception of a single proband (patient 43) harboring the homozygous p.Glu283Lys change in the choline kinase motif of the enzyme [13].

Available data about the age at last examination showed a mean age of 10.77 ± 6.30 years for group 1 compared with 20.23 ± 12.27 years for group 2 (Mann–Whitney *U* test, *p* = 0.0271).

Considering the best motor skill gained, the proportion between “walkers” and “sitters” was 76% versus 24% in group 1 and 92% versus 8% in group 2 (Fig. 3C).

Cognitive impairment (autism spectrum disorder, attention deficit hyperactivity disorder, intellectual disabilities, limited intelligence) is present in virtually all the patients with the exceptions of two Canadian siblings (patients 38 and 39) who displayed initial symptoms in the second decade with mild disease course (Fig. 3D) [10]. Intellectual disabilities seem to be less severe in subjects from group 2. Group 1 patients present more frequently autistic features compared to group 2 (70% versus 45%, respectively).

Cardiac involvement (dilated cardiomyopathy, decreased left ventricular systolic function, congenital heart defects) has been reported in 11 of 33 group 1 MCMD patients (33%) and resulted fatal in six of them [6, 8, 9, 12–15]. Heart disease was reported in a single patient (patient 35) from group 2 in the considered observation time (Fig. 3E) [9].

Broad clinical heterogeneity, observed within each group, limit the range of this genotype–phenotype correlation. For example, 2 patients from group 1 displayed symptoms onset after 5 years of age (patients 7 and 13 [13]) and carried C-term truncating mutations expected to partially preserve the choline kinase catalytic site. Marked clinical heterogeneity was also reported in the same familial group as in the case of patient 6 (onset at 14 months of age and unable to walk) and his sibling patient 7 (onset after 6 years of age and walking independently at 3 years of age) [13].

In our patient, the p.Gly354Arg change affects a conserved residue downstream the choline kinase domain in the C-term region where other pathogenic variants were previously detected. The c.448-56del27 microdeletion acts a splicing modifier, resulting in a partial intronic retention leading to a shift in the reading frame at the level of the ATP binding loop. Therefore, our case displays a mixed genotype with a null allele in trans with a missense change. Age at onset and delayed age at walking seem to indicate that this genotype causes a severe form of MCMD resembling clinical presentations of group 1 patients. Intellectual disability was present, confirming this symptom as a cardinal feature of MCMD. So far, the patient has not developed cardiomyopathy at clinical or instrumental level, but cardiac monitoring is highly recommended.

Muscle biopsy examination was fundamental to drive molecular testing and provided a valuable resource for the validation of *CHKB* defects at transcript level and the execution of biochemical studies. MCMD features typical giant mitochondria with evidence of abnormal cristae at the periphery of the fiber at electron microscopy. Histological studies also showed enlarged mitochondria at the periphery of the fibers and rarefied areas at the center. Dystrophic features and mitochondrial changes are homogenously observed in all the muscle biopsies so far available from MCMD patients. Muscle deterioration is confirmed by increased (mild to moderate) serum CK levels (90% of tested patients).

The pathogenesis of MCMD, although not entirely understood, was extensively investigated in the last 15 years [3]. Investigations performed on Chkb-ko rmd mouse showed impaired respiratory function, increased reactive oxygen species, and enhanced mitophagy in muscle tissue [7, 8]. The introduction of a muscle-specific Chkb transgene fully rescued motor and behavioral function in the rmd mouse model, confirming the cell-autonomous nature of the disease. More importantly, AAV6-based intramuscular gene therapy improved dystrophy phenotype even after disease onset in preclinical models [16]. Altered lipid metabolism was also demonstrated to result in the increase of the arrhythmogenic lipid acylcarnitine predisposing to arrhythmia in hypertrophic cardiac muscles [17]. Tavasoli and colleagues have recently demonstrated that a temporal change in lipid metabolism occurs in Chkb − / − affected muscles. They observed that impaired β-oxidation of fatty acids in mitochondria results in triacylglycerol accumulation as the disease progresses. Interestingly, the decrease in peroxisome proliferator-activated receptors (PPAR) and downstream target gene expression can be reversed by pharmacological PPAR agonism [18].

Irregular mitochondrial morphology is linked to hampered mitochondrial fission consequent to decreased levels of the fission protein DRP1, compromising OXPHOS activity [19]. Aksu‐Menges and colleagues have recently observed altered mitochondrial morphology, reduced levels of mitochondrial fission proteins and derangement in several mitochondrial pathways in human primary skeletal muscle cells from a MCMD patient [20]. Our study confirms these findings in the muscle of our patient: engaged autophagy was indirectly suggested by increased levels of p62 and LC3 in some muscle fibers while decreased levels of DRP1 were associated with a severe multi-complex defect in presence of normal levels of respiratory chain protein subunits. The rarefication of mitochondria in the center of muscle fibers, observed in our case as well as in previous reports [1, 20], might be a consequence of sustained mitophagy.

Nowadays, modern diagnostic approach based on NGS sequencing bypass the need of invasive procedures to achieve a molecular diagnosis in a relevant number of patients with neuromuscular disorders. Nevertheless, we highlight the appropriateness of muscle biopsy for the validation of genetic findings and, as in the case of MCMD, for the identification of pathognomonic features which unequivocally direct the molecular analysis.

## Conclusions

Our findings expand the genetic repertoire of MCMD and support the role of altered mitochondrial morphology and dynamics in the establishment of the severe respiratory chain defect which underline muscle pathology in this form of congenital myopathy. Additional cases and prolonged follow up of *CHKB*-mutated patients are required to challenge the genotype–phenotype correlation advanced in this report.

## Supplementary Information

**Additional file1: Supplementary Table 1.** Clinical, instrumental, histological and molecular features of CHKB-mutated MCMD patients reported so far (y: years; m: months; d: days; NA: not assessed; DCM: dilated cardiomyopathy; LVFS: left ventricular systolic function; PDA: Patent ductus arteriosus).

## Data Availability

All data generated or analyzed during this study are included in this published article.

## Abbreviations

MCMD: Megaconial congenital muscular dystrophy
EMG: Electromyography
MRI: Magnetic resonance imaging
ENT: Ear, nose, and throat evaluation
CK: Creatine kinase
COX: Cytochrome c oxidase
OXPHOS: Oxidative phosphorylation
PPAR: Peroxisome proliferator-activated receptors
