# Supplementary material for: Megaconial congenital muscular dystrophy due to novel CHKB variants: a case report and literature review
Source: Skelet Muscle. 2022 Sep 29;12:23. doi: 10.1186/s13395-022-00306-8 (PMC9524117; doi:10.1186/s13395-022-00306-8)
Supplement: Supplementary file 1 — Additional file1: Supplementary Table 1. Clinical, instrumental, histological and molecular features of CHKB-mutated MCMD patients reported so far (y: years; m: months; d: days; NA: not assessed; DCM: dilated cardiomyopathy; LVFS: left ventricular systolic function; PDA: Patent ductus arteriosus). [file 13395_2022_306_MOESM1_ESM.pdf]

		Genotype						Intellectual Disability							Muscle Biopsy			
#, Sex	Ethnic origin	Allele 1	Allele 2	Age at onset	Age at last exam.	Walk since	Best Motor	Autistic traits	Language / Cognitive impairment	MRI brain	Seizures	Heart	Skin changes	CK	Dystrophic features	Mitochondrial changes	Comment	Reference
**Group 1 (non-sense, frameshift)**																		
1, F	Japanese	c.810T>A (p.Tyr270*)	c.810T>A (p.Tyr270*)	6m	13y	2y 6m	Walker	+	+	NA	+	DCM	-	2x	+	+	Death (13y)	(Mitsuhashi and Nishino 2013; Mitsuhashi et al. 2011)
2, M	Japanese	c.810T>A (p.Tyr270*)	c.810T>A (p.Tyr270*)	5m	23y	1y 9m	Walker	+	+	Mild brain atrophy	+	DCM after 13y	-	12x	+	+	Death (23y)	(Mitsuhashi and Nishino 2013; Mitsuhashi et al. 2011)
3, F	Japanese	c.458dup (p.Leu153Phefs*57)	c.116C>A (p.Ser39*)	7m	28y	1y 6m	Walker	+	+	Cavum septum pellucidum	+	Increased cardiothoracic ratio (60%)	-	2-3x	+	+		(Mitsuhashi and Nishino 2013; Mitsuhashi et al. 2011)
4, M	Japanese (brother #3)	c.458dup (p.Leu153Phefs*57)	c.116C>A (p.Ser39*)	7m	22y	2y 6m	Walker	+	+	Normal	+	Normal	-	Normal	+	+		(Mitsuhashi and Nishino 2013; Mitsuhashi et al. 2011)
5, M	Turkish	c.611_612insC (p.Thr205Asnfs*5)	c.611_612insC (p.Thr205Asnfs*5)	4y 1m	11 y	2y 1m	Walker	+ (Severe)	+	Thin corpus callosum	+	Decreased LVSF	+	3x	+	+		(Mitsuhashi et al. 2011; Haliloglu et al. 2015; Mitsuhashi and Nishino 2013)
6, M	Turkish	c.922C>T (p.Gln308*)	c.922C>T (p.Gln308*)	14m	2y 1m	No	Sitter	-	+	Brain atrophy	-	DCM	-	Normal	+	+	Death (2y)	(Mitsuhashi et al. 2011; Haliloglu et al. 2015; Mitsuhashi and Nishino 2013)
7, M	Turkish (sibling #6)	c.922C>T (p.Gln308*)	c.922C>T (p.Gln308X*)	6y 2m	6y 6m	3y	Walker	+	+ (Moderate)	Normal	-	Normal	+	Normal	+	+	Death (7y)	Haliloglu et al. 2015
8, F	Turkish	c.677+1G>A r.spl	c.677+1G>A r.spl	3y	23y	3y	Walker	+ (Severe)	+ (Severe)	Normal	+	Atrial septal defect	+	5-6x	+	+	Lost deambulation (17y)	(Mitsuhashi et al. 2011; Haliloglu et al. 2015; Mitsuhashi and Nishino 2013)
9, F	Turkish	c.677+1G>A r.spl	c.677+1G>A r.spl	4y	9 y	No	Sitter	-	+ (Severe)	Normal	-	Normal	+	2-3x	+	+		(Mitsuhashi et al. 2011; Haliloglu et al. 2015; Mitsuhashi and Nishino 2013)
10, F	Turkish	c.677+1G>A r.spl	c.677+1G>A r.spl	3y	10y	3y	Walker	+	+ (Mild)	Delayed myelination	-	MPV	-	2-3x	+	+		(Mitsuhashi et al. 2011; Haliloglu et al. 2015; Mitsuhashi and Nishino 2013)
11, M	Turkish	c.1031+1G>A r.spl.	c.1031+1G>A r.spl	3y	11y	No	Sitter	+	+ (Mild)	Normal	-	Decreased LVSF	+	2x	+	+		(Mitsuhashi et al. 2011; Haliloglu et al. 2015; Mitsuhashi and Nishino 2013)
12, F	Turkish	c.1031+1G>A r.spl.	c.1031+1G>A r.spl	4y	12y	1y 3m	Walker	-	+ (Moderate)	Normal	-	NA	+	6x	+	+		(Mitsuhashi et al. 2011; Haliloglu et al. 2015; Mitsuhashi and Nishino 2013)
13, F	Turkish	c.922C>T (p.Gln308*)	c.922C>T (p.Gln308*)	8y	9y	3y 6m	Walker (tip-toes)	-	+ (Severe)	NA	+	Normal	+	Normal	+	+		Haliloglu et al. 2015
14, M	Turkish	c.1031+1G>A r.spl.	c.1031+1G>A r.spl	23m	4y 11m	No	Sitter	-	+ (Mild)	NA	-	NA	+	3x	+	+		Haliloglu et al. 2015
15, F	Turkish	c.1031+1G>A r.spl.	c.475C>T (p.Arg159*)	3y	8y	1y 6m	Walker	-	+	Cavum septum pellucidum	-	Normal	-	3x	+	+		Haliloglu et al. 2015
16, M	British	c.852_859del (p.Trp284*)	c.852_859del (p.Trp284*)	22m	8y	3y 4m	Walker	+	+	Normal	-	DCM (8y)	+	3-7x	+	+	Death (8y). Worsening after vaccination	(Quinlivan et al. 2013; Mitsuhashi and Nishino 2013)
17, M	African-American	c.874G>T (p.Glu292*)	c.874G>T (p.Glu292*)	11m	2y 1m	No	Sitter	+	+ (Severe)	Enlarged ventricles	+	NA	NA	3x	+	+		(Gutiérrez Ríos et al. 2012; Mitsuhashi et al. 2013)
18, M	Spanish	c.810T>A (p.Tyr270*)	c.810T>A (p.Tyr270*)	12m	13y	1y	Walker	+	+ (Mild)	Normal	-	Normal	-	2x	+	+		(Castro-Gago et al. 2014)
19, F	Portugal	c.1031+3G>C r.spl.	c.1031+3G>C r.spl	4y	22y	2y 6m	Walker	+	+	NA	-	DCM (10y)	+	2-9x	+	+		(Oliveira et al. 2015)
20, M	Spanish	c.810T>A (p.Tyr270*)	c.810T>A (p.Tyr270*)	4m	4y 6m	-	Sitter	+	+	Normal	-	Normal	+	2x	+	+		(Castro-Gago et al. 2016)
21, F	Italian	c.648C>A (p.Tyr216*)	p c.648C>A (p.Tyr216*)	Birth	9y	2y	Walker	-	+ (Severe)	Normal	-	Normal	-	7-8x	+	+		(Cabrera-Serrano et al. 2015)
22, M	Moroccan	c.248-249insT (p.Arg84Profs*209)	c.248-249insT (p.Arg84Profs*209)	16m	10y	1y 4m	Walker	-	+ (Severe)	NA	-	+	-	26-27x	+	+	Heart transplantation (10y)	(Vanlander et al. 2016)
23, F	Italian	c.565_568delTTTG (p.Leu188fs*7)	c.565_568delTTTG (p.Leu188fs*7)	Birth	11y	30m	Walker (waddling gait)	-	+ (Severe)	Normal	+	Normal	+	2x	+	+		(Marchet et al. 2019)
24, F	Italian (sister #23)	c.565_568delTTTG (p.Leu188fs*7)	c.565_568delTTTG (p.Leu188fs*7)	1m	10y	18m	Walker	+ (Severe)	+ (Severe)	NA	-	DCM (9y)	+	6x	+	+	Death (10y)	(Marchet et al. 2019)
25, F	Italian	c.140_146del (p.Arg47Profs*21)	c.1066_1067delTG (p.Trp356Valfs*72)	Birth	13y	2y	Walker (waddling gait)	+ (Severe)	+ (Severe)	NA	+	Normal	-	2x	+	+		(Marchet et al. 2019)
26, F	Chinese	c.598del (p.Gln200Argfs*11)	c.598del (p.Gln200Argfs*11)	12m	12y	5y	Walker	-	+ (Moderate)	Normal	-	Normal	-	2-3x	+	NA		Chan 2020
27, F	Chinese	c.598del (p.Gln200Argfs*11)	c.598del (p.Gln200Argfs*11)	14m	9m	1y 6m	Walker	+ (Moderate)	+ (Severe)	Normal	-	-	-	2-3x	+	+		Chan 2020
28, F	South India	c.1027dupA (p.Ser343Lysfs*86)	c.1027dupA (p.Ser343Lysfs*86)	7m	6y	2y 6m	Walker	+	+ (Severe)	NA	-	Normal	+	3-7x	NA	+		(Bardhan et al. 2021)
29, F	South India	c.224+1G>T r.spl.	c.224+1G>T r.spl.	5m	6y	No	Sitter	+	+ (Severe)	Mild brain atrophy, thin corpus callosum, enlarged ventricles	-	Normal	+	3x	NA	NA	Rett syndrome	(Bardhan et al. 2021)
30, M	South India (brother #29)	c.224+1G>T r.spl.	c.224+1G>T r.spl.	3y	3y	No	Sitter	+ (Severe)	+ (Severe)	NA	-	NA	-	NA	NA	NA	Rett syndrome	(Bardhan et al. 2021)
31, M	West of India	c.1123C>T (p.Gln375*)	c.1123C>T (p.Q375)	6m	7y	NA	Walker (with support)	+ (Severe)	+ (Severe)	NA	-	Normal	-	NA	NA	NA	Rett syndrome	(Bardhan et al. 2021)
32, F	Turkish (?)	c.818+1G>A r.spl.	c.818+1G>A r.spl.	1y	9y	4y	Walker	+	+ (Severe)	Normal	+	DCM	-	5-9x	+	+		(Kutluk et al. 2020)
33, M	Turkish (?)	c.1031+1G>A r.spl.	c.1031+1G>A r.spl.	9m	8y 6m	5y	Walker	+	+ (Mild)	Normal	-	Normal	-	5-9x	+	+		(Kutluk et al. 2020)
**Group 2 (missense, in-frame)**																		
34, M	Turkish	c.1007_1010delAGA	c.1007_1010delAGA	4y	14y 5m	1y 6m	Walker	+ (Mild)	+ (Moderate)	Enlarged ventricles	-	-	-	6x	+	+		Haliloglu et al. 2015
35, F	British	c.881C>G	c.722A>G	2y	22y	1y 1m	Walker	-	+	Normal	+	DCM (18y)	-	2x	+	+	Lost Deambulation (21y)	(Quinlivan et al. 2013; Mitsuhashi and Nishino 2013)

		(p.Pro294Arg)	(p.Asn241Ser)															
36, M	British	c.722A>G (p.Asn241Ser)	c.722A>G (p.Asn241Ser)	3y	3y	2y 6m	Walker	NA	+ (Mild)	Normal	-	-	+	5x	+	+		(Quinlivan et al. 2013; Mitsuhashi and Nishino 2013)
37, M	French	c.581G>A (p.Arg194Gln)	c.581G>A (p.Arg194Gln)	2y	NA	2y	Walker	+	+	NA	-	NA	NA	Raised	+	+		(Mitsuhashi and Nishino 2013)
38, F	Canadian	c.950T>A (p.Leu317Gln)	c.263C>T (p.Pro88Leu)	16y	40y	Normal	Walker	-	-	NA	-	Normal	-	5-40x	+	+	Myalgias, worsening after stress/illness	(Brady et al. 2016)
39, M	Canadian (brother #36)	c.950T>A (p.Leu317Gln)	c.263C>T (p.Pro88Leu)	15y	36y	Normal	Walker	-	-	NA	-	Normal	-	40 x	+	+	Rhabdomyolysis occasional	(Brady et al. 2016)
40, M	Indian	c.722A>G (p.Asn241Ser)	c.722A>G (p.Asn241Ser)	6m	11y	1y 1m	Walker	-	+ (Severe)	Normal	-	-	-	5-6x	+	+	Onset after illness	(De Goede et al. 2016)
41, F	Bulgarian	c.701C>T (p.Ser234Leu)	c.701C>T (p.Ser234Leu)	16y	35y	1y	Walker	-	+ (Mild)	Normal	-	-	-	8-9x	+	+		(De Fuenmayor-Fernández De La Hoz et al. 2016)
42, F	South India	c.581G>A (p.Arg194Gln)	c.581G>A (p.Arg194Gln)	5y	12y	2y	Walker	-	+ (Mild)	Normal	-	Normal	No	5-13x	+	+		(Bardhan et al. 2021)
43, F	Turkish	c.847G>A (p.Glu283Lys)	c.847G>A (p.Glu283Lys)	38d	8y 2m	No	Sitter	-	+	Normal	-	- (PDA)	-	1-2 x	+	+		Haliloglu et al. 2015
44, M	Turkish	c.1130G>T (p.Arg377Leu)	c.1130G>T (p.Arg377Leu)	1y	18y	2y	Walker	-	+ (Mild)	NA	-	-	-	5-6x	+	+		(Mitsuhashi et al. 2011; Haliloglu et al. 2015; Mitsuhashi and Nishino 2013)
45, F	Turkish	c.554_562del (p.Pro185_Trp187del)	c.554_562del (p.Pro185_Trp187del)	17y	23y	3y	Walker	+ (Severe)	+ (Severe)	NA	+	-	-	13x	+	+		(Mitsuhashi et al. 2011; Haliloglu et al. 2015; Mitsuhashi and Nishino 2013)

Supplementary Table 1

Clinical, instrumental, histological and molecular features of *CHKB*-mutated MCMD patients reported so far (y: years; m: months; d: days; NA: not assessed; DCM: dilated cardiomyopathy; LVFS: left ventricular systolic function; PDA: Patent ductus arteriosus).

Supplemental Data

Supplementary Materials and Methods

Histological and immunohistochemical analysis

Tissue specimen was frozen in isopentane-cooled liquid nitrogen and processed according to standard techniques. For histological analysis, 8 μm-thick cryosections were picked and processed for routine staining with Haematoxylin and Eosin (H&E), Modified Gomori Trichrome (MGT), myosin ATPase (pH 9.4-4.6-4.3), cytochrome c oxidase (COX), succinate dehydrogenase (SDH), phosphatase acid, NADH, Oil Red O, Periodic Acid Schiff (PAS).

Immunohistochemical analyses were performed on 8-μm-thick muscle cryosections after fixation with cold acetone for 3 min. After blocking with 1% BSA in PBS and permeabilization with 0.1% TritonX-100 in PBS, sections were incubated with primary antibodies: anti-caveolin-3 (BD Transduction, Franklin Lakes, NJ, USA), anti-p62/SQSTM1 (Abcam, Cambridge, UK), anti-LC3A (ThermoFisher, Waltham, MA, USA), anti-dystrophin Rod domain (NCL-DYS1), anti-dystrophin C-terminus (NCL-DYS2), anti-dystrophin NH2-terminus, anti-SG-alpha, SG-gamma, all from Novocastra Leica Biosystem, Newcastle upon Tyne, UK After washing, sections were incubated with secondary Alexa Fluor 488-labeled goat anti rabbit and Alexa Fluor 568-labeled goat anti mouse. Sections were mounted in antifade mounting medium with DAPI (Vectashield Vibrance, Vector Laboratories, Burlingame, CA, USA). Images fields were acquired at 40X using optical microscope Leica DM4000B equipped with DFC420C camera.

Electron microscopy

For ultrastructural examination a small part of muscle sample was fixed in 2.5% glutaraldehyde (pH 7.4), post fixed in 2% osmium tetroxide and then, after dehydration in a graded series of ethanol, embedded in Epon's resin. Finally, ultrathin sections were stained with lead citrate and uranyl acetate and examined with Zeiss EM109 transmission electron microscope.

Molecular studies

Genomic DNA was extracted from peripheral blood samples according to standard procedures. *CHKB* coding regions and intronic boundaries were re-sequenced for molecular testing and segregation studies by Sanger sequencing on an ABI Prism 3130 platform.

mRNA was isolated from tissues and cells with Eurozol. Then, cDNA was produced through reverse transcription polymerase chain reaction (RT-PCR) using the RT Maxima Reverse Transcription Master Mix (Thermo Fisher). RT-PCR amplicons were electrophoresed on agarose gels

before direct seqeucning. Cloning experiments were performed by using TOPO-TA Cloning system (Life Technologies). All the primers are available upon request.

Biochemical studies

Levels of MFN2 and DRP1 proteins were evaluated in total lysates of muscle in a 4-12% SDS-PAGE by using appropriate monoclonal antibodies (MFN-2: D1E9 11925S Cell Signalling 1:1000; DRP-1 abcam ab56788 1:500). Licor Odissey FC was used for image acquisition and analysis. The amount of proteins was detected using florescent secondary antibodies (LI-COR IR-DYE 800 -680 CW). Actin (Sigma A2066) was used for normalization purpose. A cocktail of antibodies was used to assess mitochondrial respiratory chain subunits (abcam ab110411, 1:1000). The mitochondrial PORIN (VDAC) was assayed by using a specific antibody (abcam ab15895, 1:1500).

Mitochondrial respiratory chain enzyme and citrate synthase activities were measured spectrophotometrically as previously described (Galbiati et al., 2006). The specific activity of each complex was normalized to that of citrate synthase.

Supplementary References

- Bardhan M, Polavarapu K, Bevinahalli NN, Veeramani PK, Anjanappa RM, Arunachal G, Shingavi L, Vengalil S, Nashi S, Chawla T, Nagabushana D, Mohan D, Horvath R, Nishino I, Atchayaram N. Megaconial congenital muscular dystrophy secondary to novel CHKB mutations resemble atypical Rett syndrome. J Hum Genet. 2021 Aug;66(8):813-823. doi: 10.1038/s10038-021-00913-1. Epub 2021 Mar 12. Erratum in: J Hum Genet. 2021 Mar 26;: PMID: 33712684.
- Brady L, Giri M, Provias J, Hoffman E, Tarnopolsky M. Proximal myopathy with focal depletion of mitochondria and megaconial congenital muscular dystrophy are allelic conditions caused by mutations in CHKB. Neuromuscul Disord. 2016 Feb;26(2):160-4. doi: 10.1016/j.nmd.2015.11.002. Epub 2015 Dec 4. PMID: 26782016.
- Cabrera-Serrano M, Junckerstorff RC, Atkinson V, Sivadorai P, Allcock RJ, Lamont P, Laing NG. Novel CHKB mutation expands the megaconial muscular dystrophy phenotype. Muscle Nerve. 2015 Jan;51(1):140-3. doi: 10.1002/mus.24446. Epub 2014 Nov 22. PMID: 25187204.
- Castro-Gago M, Dacruz-Alvarez D, Pintos-Martínez E, Beiras-Iglesias A, Arenas J, Martín MÁ, Martínez-Azorín F. Congenital neurogenic muscular atrophy in megaconial myopathy due to a mutation in CHKB gene. Brain Dev. 2016 Jan;38(1):167-72. doi: 10.1016/j.braindev.2015.05.008. Epub 2015 May 23. PMID: 26006750.
- Chan SH, Ho RS, Khong PL, Chung BH, Tsang MH, Yu MH, Yeung MC, Chan AO, Fung CW. Megaconial congenital muscular dystrophy: Same novel homozygous mutation in CHKB gene in two unrelated Chinese patients. Neuromuscul Disord. 2020 Jan;30(1):47-53. doi: 10.1016/j.nmd.2019.10.009. Epub 2019 Nov 5. PMID: 31926838.
- De Fuenmayor-Fernández De La Hoz CP, Domínguez-González C, Gonzalo-Martínez JF, Esteban-Pérez J, Fernández-Marmiesse A, Arenas J, Martín MA, Hernández-Laín A. A milder phenotype of megaconial

congenital muscular dystrophy due to a novel CHKB mutation. Muscle Nerve. 2016 Oct;54(4):806-8. doi: 10.1002/mus.25183. Epub 2016 Jun 9. PMID: 27169979.

- De Goede C, Oh T, Joseph J, Muntoni F, Sewry C, Phadke R. Choline Kinase Beta-Related Muscular Dystrophy, Appearance of Muscle Involvement on Magnetic Resonance Imaging. Pediatr Neurol. 2016 Jan;54:49-54. doi: 10.1016/j.pediatrneurol.2015.09.018. Epub 2015 Nov 6. PMID: 26548592.
- Galbiati S, Bordoni A, Papadimitriou D, Toscano A, Rodolico C, Katsarou E, Sciacco M, Garufi A, Prelle A, Aguennouz M', Bonsignore M, Crimi M, Martinuzzi A, Bresolin N, Papadimitriou A, Comi GP. New mutations in TK2 gene associated with mitochondrial DNA depletion. Pediatr Neurol. 2006 Mar;34(3):177-85. doi: 10.1016/j.pediatrneurol.2005.07.013. PMID: 16504786.
- Gutiérrez Ríos P, Kalra AA, Wilson JD, Tanji K, Akman HO, Area Gómez E, Schon EA, DiMauro S. Congenital megaconial myopathy due to a novel defect in the choline kinase Beta gene. Arch Neurol. 2012 May;69(5):657-61. doi: 10.1001/archneurol.2011.2333. PMID: 22782513; PMCID: PMC8276349.
- Haliloglu G, Talim B, Sel CG, Topaloglu H. Clinical characteristics of megaconial congenital muscular dystrophy due to choline kinase beta gene defects in a series of 15 patients. J Inherit Metab Dis. 2015 Nov;38(6):1099-108. doi: 10.1007/s10545-015-9856-2. Epub 2015 Jun 12. PMID: 26067811.
- Kutluk G, Kadem N, Bektas O, Eroglu HN. A Rare Cause of Autism Spectrum Disorder: Megaconial Muscular Dystrophy. Ann Indian Acad Neurol. 2020 Sep-Oct;23(5):694-696. doi: 10.4103/aian.AIAN_98_19. Epub 2020 Dec 8. PMID: 33623274; PMCID: PMC7887486.
- Mitsuhashi S, Nishino I. Phospholipid synthetic defect and mitophagy in muscle disease. Autophagy. 2011 Dec;7(12):1559-61. doi: 10.4161/auto.7.12.17925. PMID: 22024749; PMCID: PMC3288031.
- Mitsuhashi S, Ohkuma A, Talim B, Karahashi M, Koumura T, Aoyama C, Kurihara M, Quinlivan R, Sewry C, Mitsuhashi H, Goto K, Koksal B, Kale G, Ikeda K, Taguchi R, Noguchi S, Hayashi YK, Nonaka I, Sher RB, Sugimoto H, Nakagawa Y, Cox GA, Topaloglu H, Nishino I. A congenital muscular dystrophy with mitochondrial structural abnormalities caused by defective de novo phosphatidylcholine biosynthesis. Am J Hum Genet. 2011 Jun 10;88(6):845-851. doi: 10.1016/j.ajhg.2011.05.010. PMID: 21665002; PMCID: PMC3113344.
- Oliveira J, Negrão L, Fineza I, Taipa R, Melo-Pires M, Fortuna AM, Gonçalves AR, Froufe H, Egas C, Santos R, Sousa M. New splicing mutation in the choline kinase beta (CHKB) gene causing a muscular dystrophy detected by whole-exome sequencing. J Hum Genet. 2015 Jun;60(6):305-12. doi: 10.1038/jhg.2015.20. Epub 2015 Mar 5. PMID: 25740612.
- Quinlivan R, Mitsuahashi S, Sewry C, Cirak S, Aoyama C, Mooore D, Abbs S, Robb S, Newton T, Moss C, Birchall D, Sugimoto H, Bushby K, Guglieri M, Muntoni F, Nishino I, Straub V. Muscular dystrophy with large mitochondria associated with mutations in the CHKB gene in three British patients: extending the clinical and pathological phenotype. Neuromuscul Disord. 2013 Jul;23(7):549-56. doi: 10.1016/j.nmd.2013.04.002. Epub 2013 May 18. PMID: 23692895.
- Vanlander AV, Muiño Mosquera L, Panzer J, Deconinck T, Smet J, Seneca S, Van Dorpe J, Ferdinande L, Ceuterick-de Groote C, De Jonghe P, Van Coster R, Baets J. Megaconial muscular dystrophy caused by mitochondrial membrane homeostasis defect, new insights from skeletal and heart muscle analyses. Mitochondrion. 2016 Mar;27:32-8. doi: 10.1016/j.mito.2016.02.001. Epub 2016 Feb 23. PMID: 26855408.
